# Characterization of lignin derived from water-only and dilute acid flowthrough pretreatment of poplar wood at elevated temperatures

**DOI:** 10.1186/s13068-015-0377-x

**Published:** 2015-12-01

**Authors:** Libing Zhang, Lishi Yan, Zheming Wang, Dhrubojyoti D. Laskar, Marie S. Swita, John R. Cort, Bin Yang

**Affiliations:** Bioproduct Sciences and Engineering Laboratory, Department of Biological Systems Engineering, Washington State University, Richland, WA 99354 USA; Fundamental and Computational Sciences Directorate, Pacific Northwest National Laboratory, Richland, WA 99354 USA; Bioproduct Sciences and Engineering Laboratory, Pacific Northwest National Laboratory, Richland, WA 99354 USA

**Keywords:** Hot water, Dilute acid, Flowthrough pretreatment, Poplar, Lignin, Characterization

## Abstract

**Background:**

Flowthrough pretreatment of biomass is a critical step in lignin valorization via conversion of lignin derivatives to high-value products, a function vital to the economic efficiency of biorefinery plants. Comprehensive understanding of lignin behaviors and solubilization chemistry in aqueous pretreatment such as water-only and dilute acid flowthrough pretreatment is of fundamental importance to achieve the goal of providing flexible platform for lignin utilization.

**Results:**

In this study, the effects of flowthrough pretreatment conditions on lignin separation from poplar wood were reported as well as the characteristics of three sub-sets of lignin produced from the pretreatment, including residual lignin in pretreated solid residues (ReL), recovered insoluble lignin in pretreated liquid (RISL), and recovered soluble lignin in pretreatment liquid (RSL). Both the water-only and 0.05 % (w/w) sulfuric acid pretreatments were performed at temperatures from 160 to 270 °C on poplar wood in a flowthrough reactor system for 2–10 min. Results showed that water-only flowthrough pretreatment primarily removed syringyl (S units). Increased temperature and/or the addition of sulfuric acid enhanced the removal of guaiacyl (G units) compared to water-only pretreatments at lower temperatures, resulting in nearly complete removal of lignin from the biomass. Results also suggested that more RISL was recovered than ReL and RSL in both dilute acid and water-only flowthrough pretreatments at elevated temperatures. NMR spectra of the RISL revealed significant β-O-4 cleavage, α-β deoxygenation to form cinnamyl-like end groups, and slight β-5 repolymerization in both water-only and dilute acid flowthrough pretreatments.

**Conclusions:**

Elevated temperature and/or dilute acid greatly enhanced lignin removal to almost 100 % by improving G unit removal besides S unit removal in flowthrough system. Only mild lignin structural modification was caused by flowthrough pretreatment. A lignin transformation pathway was proposed to explain the complexity of the lignin structural changes during hot water and dilute acid flowthrough pretreatment.

**Graphical abstract:**
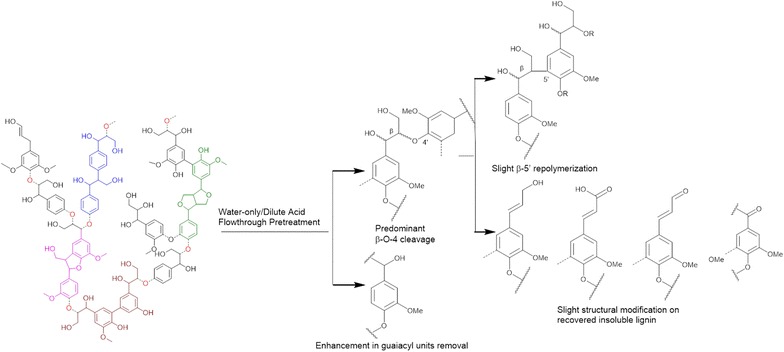
Lignin transformations in water-only and dilute acid flowthrough pretreatment at elevated temperatures

**Electronic supplementary material:**

The online version of this article (doi:10.1186/s13068-015-0377-x) contains supplementary material, which is available to authorized users.

## Background

Lignin is a main constituent of lignocellulosic biomass (15–30 % by weight, up to 40 % by energy) and the second most abundant biopolymer on Earth. Recently, lignin has received broad attention as its intermediates have found wide use in various carbon products, such as electrodes, carbon fibers, jet fuels, biochemicals, plastics, and antioxidants [1–7]. Thus, the utilization of waste lignin in pretreatment as a feedstock for conversion to fuels and chemicals offers a promising opportunity for enhancing the overall operational efficiency, carbon conversion, economic viability, and sustainability of biorefineries.

Lignin is an amorphous, cross-linked biopolymer that consists of three phenylpropanoid units, namely *p*-hydroxyphenyl (H units), guaiacyl (G units), and syringyl (S units). These units are derived from the three monolignol building blocks: *p*-coumaryl, coniferyl, and sinapyl alcohols. The major interunit linkages in lignin are β–O–4, α–O–4, β–5, β–β, 5–5, β–1, and 4–O–5 [8]. Selective depolymerization of lignin is important to lignin utilization and also considered to be very challenging due to the broad distribution of bond energies in the various C–O and C–C linkages within the structure of lignin and the tendency for uncontrollable thermal and catalytic fragmentation when trying to cleave them [4]. Most lignin extraction processes usually cause many lignin structural changes and modifications. As removing lignin is favorable to the process of enzymatic hydrolysis for sugar conversion, great efforts have been made to boost lignin extraction from the solid biomass to the liquid phase during the aqueous pretreatment [9]. Most water-only and dilute acid batch pretreatments were reported to achieve limited lignin removal [10, 11] dictated by the lignin depolymerization and condensation chemistry [12–14]. Under acidic pretreatment conditions, the predominant reactions associated with lignin are fragmentation by acidolysis of aryl ether linkages and acid-catalyzed recondensation, while linkages like resinol and phenylcoumaran sub-units are fairly stable. The β–O–4 linkages in lignin are susceptible to acidic hydrolysis, and the pretreatments generally result in their lower relative content in the pretreated biomass [15]. The dilute acid pretreatment leads to an increase of phenolic OH groups in lignin apparently resulting from cleavage of aryl ether linkages [16–18]. In addition, lignin in different biomass species has various characteristics, which influence pretreatment sugar yield and enzymatic hydrolysis effectiveness. It was reported that sugar yield was related to lignin content and structural characteristics, especially S/G ratio in the biomass substrate [19]. Also, changes in cellulose structure were influenced by lignin content [20]. Biomass genetic modification and manipulation were performed to reduce lignin recalcitrance, modify S/G ratio, and improve sugar yields [21–24]. However, biomass pretreatment is still challenging for high sugar recovery with maximum usable lignin recovery since sugars are easily degraded under high pretreatment severities. High yields of sugars and lignin are critical to achieve favorable pretreatment economics [25].

Sugar yields and removal of lignin by pretreatment can be greatly improved by flowing hot water through the biomass [25, 26]. The use of a flowthrough reactor system was reported to increase lignin removal as much as 98 % [25], and the addition of dilute acid further increased the removal extent [25, 27–29]. The main advantage of a flowthrough reactor system is its ability to restrict condensation reactions by constantly removing lignin into the aqueous phase and reducing the chance of pseudo-lignin formation [30, 31] and lignin deposition [32]. In addition, pretreatment was shown to mitigate lignin droplets on the cellulose surface [33], thus it led to effective lignin deconstruction and hemicellulose recovery [34, 35]. In pretreatment, lignin is believed to depolymerize via both homolytic and acidolytic cleavage into low-molecular weight lignin globules [15, 36]. Thus, solubilized lignin derivatives continuously exit the flowthrough system, thus further thermal or chemical treatments on depolymerized lignin fractions can be avoided. In this case, flowthrough-derived lignin is believed to only exhibit mild structural change compared to the native original lignin. Effective recovery of sugars and lignin with whole biomass solubilization by flowthrough pretreatment at elevated temperatures under tested pretreatment conditions was previously reported [25]. However, application of lignin derivatives to produce added value products cannot be realized unless the corresponding lignin recovery yield and chemistry under these pretreatment conditions are well understood. In this study, the three lignin sub-sets, including residual lignin in pretreated solid residues (ReL), the purity of recovered insoluble lignin in pretreated liquid (RISL), and recovered soluble lignin in pretreatment liquid (RSL) from flowthrough pretreatment, were measured following acetyl bromide treatment, and their structure was analyzed using Fourier transform infrared (FTIR) spectroscopy, GC/MS, pyrolysis–GC/MS, and two-dimensional (2D) ^1^H–^13^C heteronuclear single-quantum coherence (HSQC) NMR spectroscopy. Structural characteristics of three lignin isolates were investigated in order to reveal intrinsic structural modification of lignin under the tested pretreatment conditions, which will benefit the utilization of these lignin sources in the context of a biorefinery dependent on high yields of sugars.

## Results and discussion

**Fig. 1:**
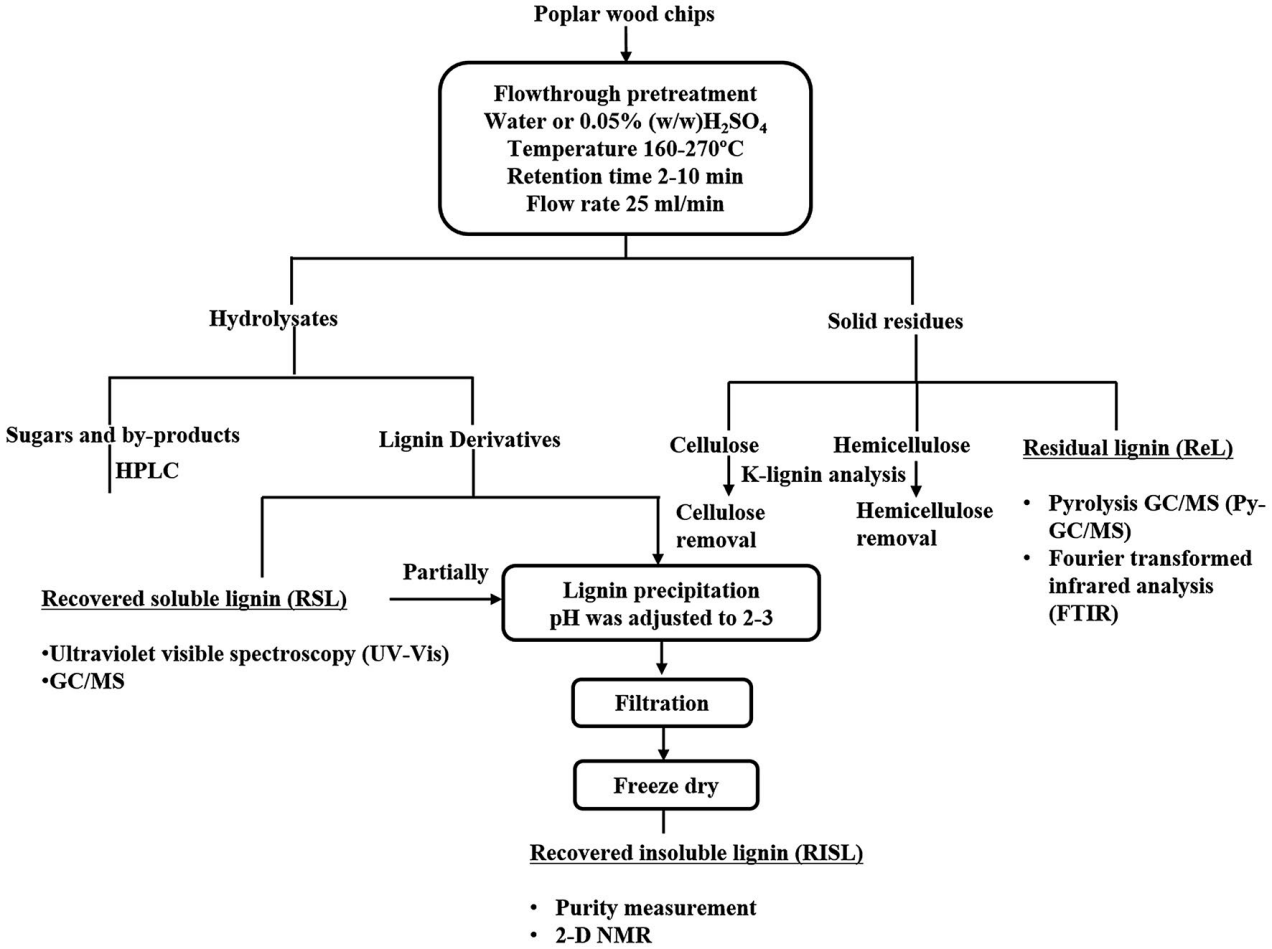
Scheme for lignin recovery and analytical analysis in this study

In this study, poplar carbohydrates were mostly recovered as sugars under tested pretreatment conditions [25, 29]. Results showed that flowthrough pretreatment at elevated temperatures enhanced sugar recovery to over 90 % and limited degradation loss. This paper focuses on understanding lignin recovery and structural characteristics of the recovered lignin, which are crucial for conversion to high-value products. To characterize the recovery of the three lignin sub-sets, a comprehensive process was followed as summarized in Fig. 1. Recovered insoluble lignin was collected by settling pretreatment liquid at pH 2–3 overnight to precipitate solid lignin at the bottom followed by fast filtration to collect lignin solids, avoiding filter paper clogging.

### Lignin removal and recovery in aqueous phase

**Fig. 2:**
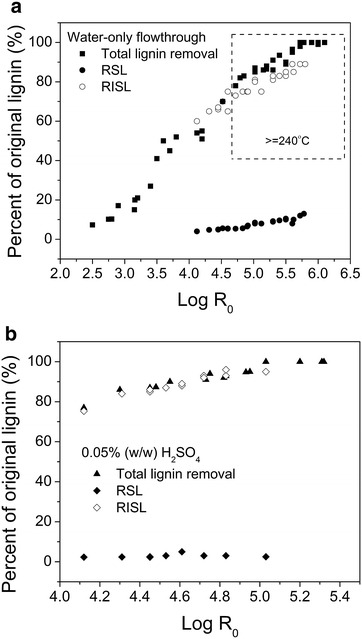
Lignin removal and recovery as RSL and RISL. **a** Water-only and **b** 0.05 % (w/w) H_2_SO_4_ flowthrough pretreatment (temperature: 160–270 °C, time: 0–12 min, flowrate: 25 ml/min)

Lignin in its pretreated aqueous phase was recovered as the RSL and RISL. Lignin recovery by flowthrough pretreatment increased as the pretreatment severity increased (Log *R*_0_) under both water-only and dilute acid conditions (Fig. 2). The pretreatment severity (Log *R*_0_) was used to evaluate the severity of the pretreatment process by combining two variables, the temperature and reaction time (Eq. 1 in the method section). Nearly 100 % of lignin was released into the aqueous phase at around Log *R*_0_ = 6.0 in water-only pretreatment or at Log *R*_0_ = 5.0 in dilute acid pretreatment. Under such conditions, it was previously reported that nearly whole biomass was solubilized into the liquid phase and carbohydrates were almost completely recovered as monomeric and oligomeric sugars [25].

Dilute acid was found to be more effective for lignin recovery than water-only pretreatment at the same severities (Fig. 2). Addition of 0.05 % (w/w) sulfuric acid enhanced delignification and solubilization into the aqueous phase. Considering that the pH of the water at 220 °C was 5.5 and that it dropped to 3.2–4.0 at temperatures higher than 220 °C [25, 29], water-only pretreatment at increased temperatures is similar to acid-catalyzed pretreatments. Different RSL and RISL yields were observed in water-only and dilute acid pretreatments (Fig. 2a, b). The RSL yield was less than 20 and 5 % in water-only and dilute acid pretreatments, respectively. Higher yields of RSL were found in water-only than in dilute acid pretreatment at the same Log *R*_0_. In addition, the dilute acid-derived RSL was observed to gradually increase with increasing Log *R*_0_, followed by a slight decline at about Log *R*_0_ = 4.6, which differed from the climbing trend of RSL in water-only pretreatment. These suggested that reactions shifted from lignin depolymerization to counterproductive RSL condensation or repolymerization in dilute acid pretreatment, thereby limiting the removal of depolymerized lignin derivatives. Since flowthrough pretreatment possibly limited the number of condensation reactions, a slight decrease in RSL yield was observed (Fig. 2b). More than 60 % of the recovered RISL was found in flowthrough pretreatment at Log *R*_0_ >4.0, and dilute acid addition further improved RISL formation. The pretreatment severities at ~6.0 and ~5.0 appeared to be the most promising conditions tested to produce high yield of RISL as well as high sugar yields by water-only and dilute acid pretreatments, respectively [25]. The lignin analyzed in this study was extracted under these two conditions.

### Lignin purity determination of the isolated RISL

**Fig. 3:**
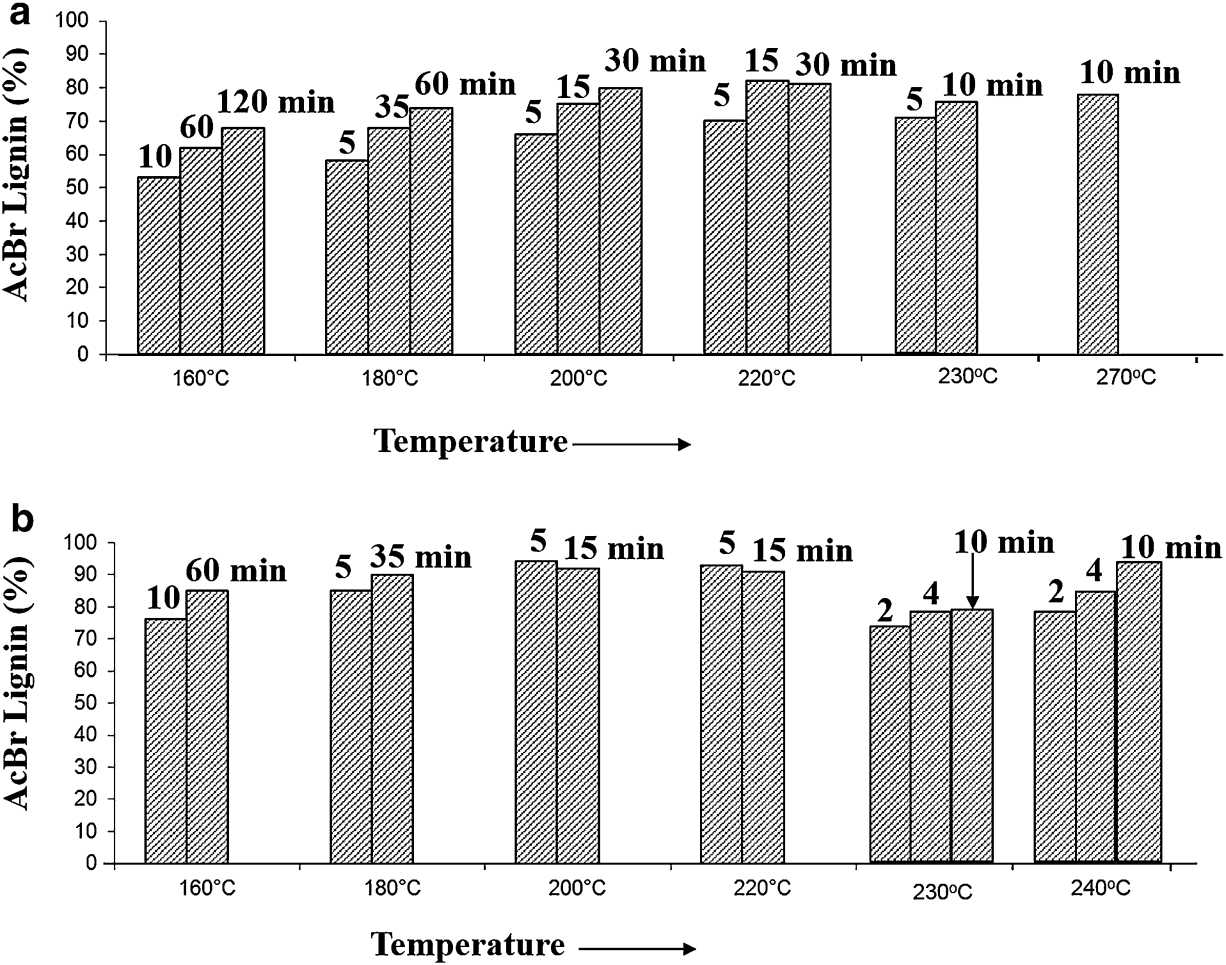
The RISL purity determined by AcBr method. **a** Water-only; **b** 0.05 % (w/w) H_2_SO_4_ at the flow rate of 25 ml/min

The flowthrough pretreatment hydrolysates contained a mixture of degradation products from carbohydrates and lignin derivatives. The RISL was isolated by precipitating hydrolysates at pH 2–3, followed by filtration, washing, and freeze-drying. The RISL purity was found to be higher than 50 % in both water-only and dilute acid pretreatment hydrolysates under the tested conditions (Fig. 3). The highest purity of RISL was achieved at about 80 and 90 % in water-only and dilute acid pretreatments, respectively. RISL pretreated using dilute acid was found to have higher purity than that in water-only pretreatment. This was because most carbohydrates were hydrolyzed into monomeric sugars and washed away in dilute acid flowthrough pretreatment [25].

RISL recovered at high temperatures (240 and 270 °C for wt 0.05 % (w/w) sulfuric acid and hot water pretreatments, respectively) was treated with pyridine/acetic anhydride. THF was used as an eluent. Individual lignin samples were dissolved in THF. GPC analysis was carried out in an Agilent GPC system with a UV detector. The set flow rate was 1 ml/min. Polystyrene was used for calibration curve. The number average molecular weight (M_n_) and the weight average molecular weight (*M*_w_) for lignin obtained at 240 °C, residence time 10 min, 0.05 % sulfuric acid, and flow rate 25 ml/min were 1083 and 1955 Da, respectively (Additional file 1: Table S1). However, *M*_n_ and *M*_w_ for lignin obtained at 270 °C, residence time 10 min, water-only condition, and flow rate 25 ml/min were 1197 and 2661 Da, respectively (Additional file 1: Figure S1). The polydispersity (*D*; *M̅*_w_/*M̅*_n_) of lignin derived from dilute acid condition (240 °C, 10 min, 0.05 % sulfuric acid, and flow rate 25 ml/min,) and hot water condition (270 °C, 10 min, water-only, and flow rate 25 ml/min) was 1.81 and 2.22, respectively.

### Py-GC/MS spectroscopic analysis of untreated poplar wood and pretreated solid residues

**Fig. 4:**
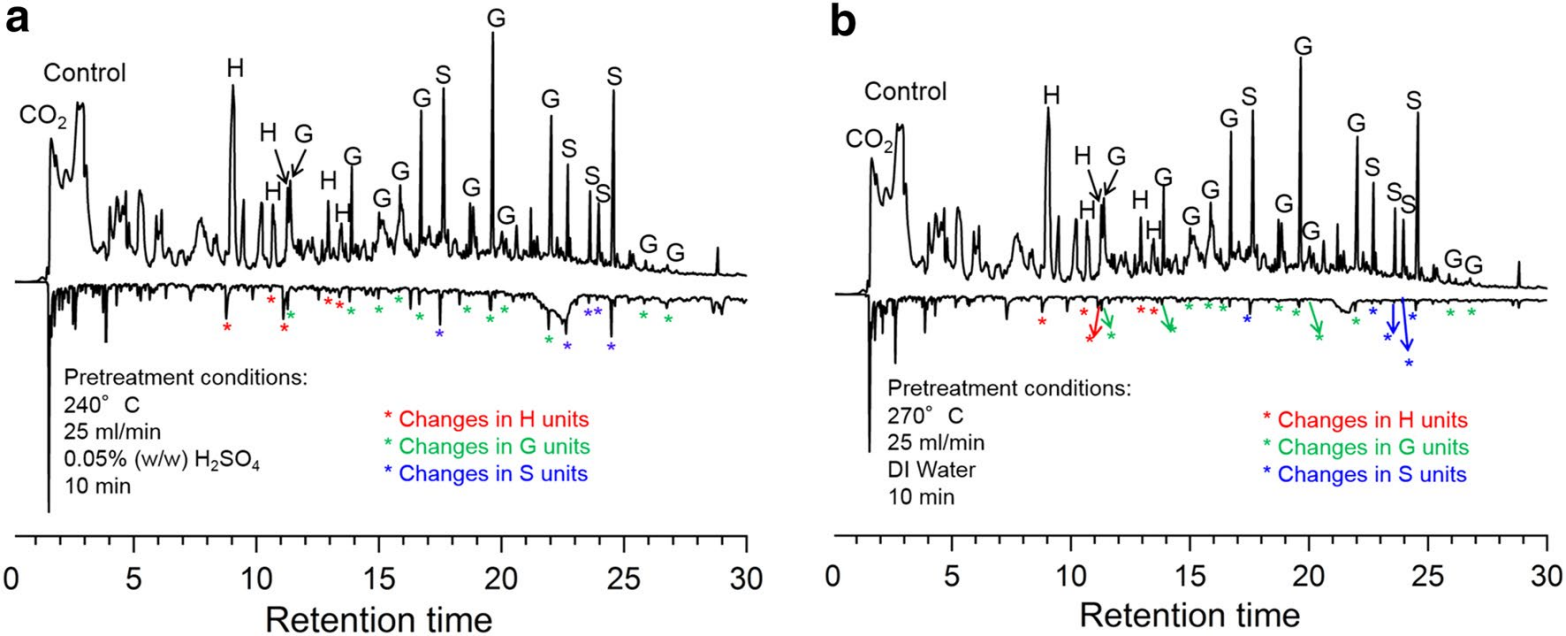
Py-GC/MS results of untreated poplar wood (control) and solid residues after flowthrough pretreatments. **a** Untreated poplar wood and solid residues pretreated at 240 °C, 0.05 % (w/w) H_2_SO_4_ for 10 min (pretreatment severity ~5.0); **b** untreated poplar wood and solid residues pretreated at 270 °C, water-only for 10 min (pretreatment severity ~6.0)

Py-GC/MS analysis of ReL revealed structural information in the form of pyrolytic products. Selected Py-GC/MS results of residual solids pretreated at 240 °C with 0.05 % (w/w) H_2_SO_4_ for 10 min (Log *R*_0_ ~ 5.0) and at 270 °C by water-only for 10 min (Log *R*_0_ ~6.0) are shown in Fig. 4. As seen from the differences among peak intensities in Fig. 4, solid residues after pretreatment showed much less lignin remaining than that in the untreated poplar wood due to the enhanced lignin depolymerization.

**Fig. 5:**
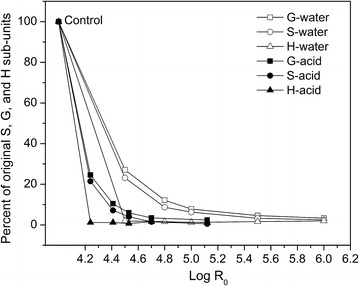
Effects of pretreatment severity on S, G, and H unit removal by water-only and 0.05 % (w/w) dilute acid pretreatment (poplar wood control sample: G:S:H = 7:11:2)

The ratios of the S, G, and H units contain structural information about lignin. The S/G ratio was used as a criterion for evaluating delignification [36, 37]. Py-GC/MS was applied to investigate S/G ratio change during flowthrough pretreatment. The preferential release of sub-units (G and S) of lignin into the aqueous phase was reported in flowthrough pretreatment at temperatures up to 200 °C [38]. In this study, nearly complete lignin removal was achieved under severities of higher than ~5.0 and ~6.0 in dilute acid and water-only pretreatments, respectively. The dynamic changes of the S, G, and H units during flowthrough pretreatment are shown in Fig. 5. The spectra in Py-GC/MS results were normalized, and the relative percentages of the S, G, and H units or their reduction based on untreated poplar lignin are shown.

Figure 5 shows the high extent of release of S, G, and H units into the aqueous phase at severities higher than 4.2 and 4.5 in dilute acid and water-only pretreatments, respectively. The removal rate of the S unit was higher than that of the G units in flowthrough pretreatment, indicating the preferential removal of S units versus G units. Such preference was consistent with previous results of water-only pretreatment at temperatures lower than 200 °C [37, 38]. The H unit, constituting about 10 % of lignin in untreated poplar wood, showed the fastest removal, which might be due to greater reactivity at the C3 and C5 positions. The elevated temperatures and addition of 0.05 % (w/w) H_2_SO_4_ enhanced lignin depolymerization, thus it not only led to removal of the S units but also promoted solubilization of the G unit.

### FTIR spectroscopic analysis of ReL

**Fig. 6:**
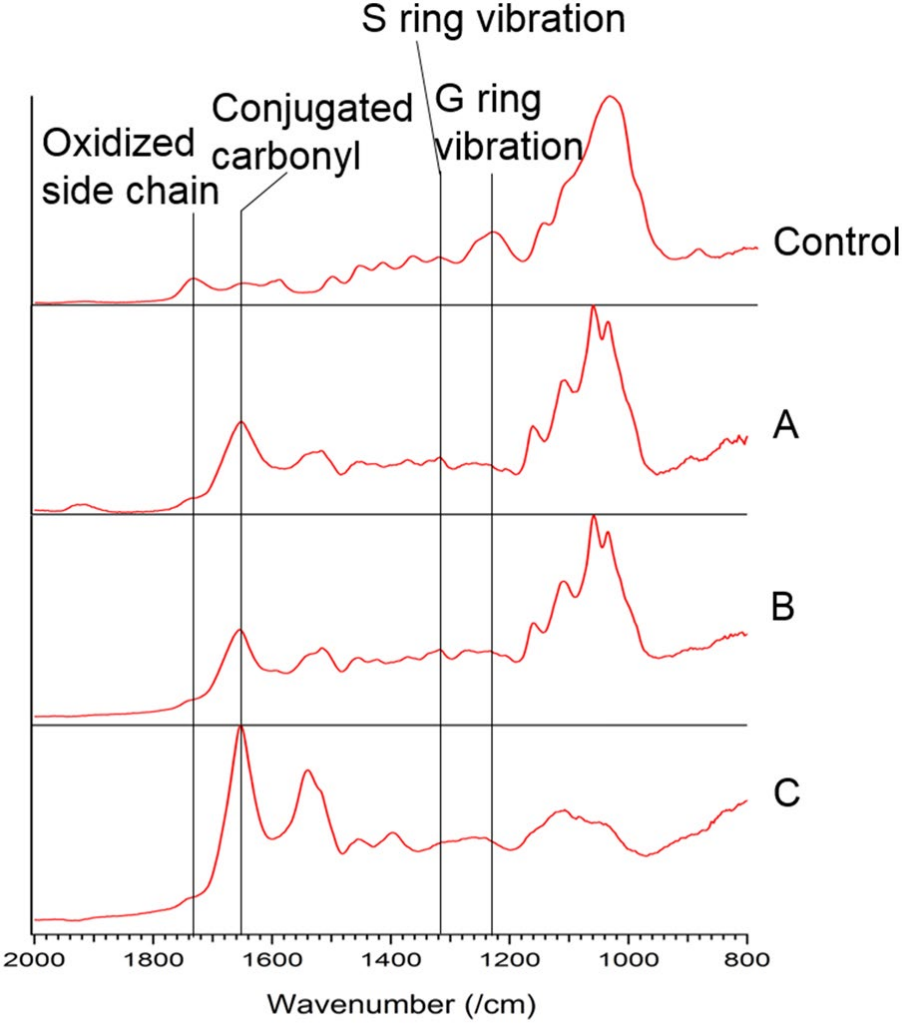
Characterization of ReL in pretreated solid residues by FTIR (800–2000 cm^−1^). Control: untreated poplar wood;* A* solid residues from 0.05 % (w/w) sulfuric acid pretreatment at 240 °C for 2.6 min;* B* solid residues from water-only pretreatment at 230 °C for 3.8 min;* C* solid residues pretreated with 0.05 % sulfuric acid at 240 °C for 5 min

FTIR was carried out to evaluate ReL structural modification in functional groups by flowthrough pretreatment. The assignments of the functional groups of lignin in this study are shown in Table 1. Peaks at 1260–1760 cm^−1^ were assigned to lignin structural spectral vibration regions [37]. In the selected spectra of solid residues obtained from both flowthrough pretreatments, ~80 % reduction of peak intensity at 1594, 1455, and 1423 cm^−1^ was observed, indicating the removal of the aromatic rings to liquid phase (Fig. 6). The shape and intensity changes in the peak at 1115 cm^−1^ were also observed to correspond with the modification of the aromatic ring and cleavage of the ester linkages, which was especially obvious in sample C (solid residues by 0.05 % sulfuric acid pretreatment at 240 °C for 5 min). The peak at 1735 cm^−1^ disappeared in all samples, which indicated the removal of oxidized side chains. In addition, the peak intensity at 1655 cm^−1^ increased, indicating the formation of conjugated carbonyl groups [37, 39], particularly under acidic conditions.

**Table 1:** FTIR spectra band assignments [47–53]

No.	Wavenumber (cm^−1^)	Assignment
1	1708–1738	Stretching of C=O unconjugated to aromatic rings (oxidized side chains, non-conjugated carbonyl)
2	1655	Stretching of C=O conjugated to aromatic rings (conjugated carbonyl)
3	1590–1609	Aromatic ring vibrations and C=O stretching
4	1500–1515	Aromatic ring vibrations
5	1455/1425	C–H deformation and aromatic ring vibration
6	1420–1424	Aromatic ring vibrations
7	1375	C–H stretching in cellulose and hemicellulose
8	1330–1325	Syringyl nuclei (C–O stretching)
9	1270-1268/1244	Guaiacyl nuclei (C–O stretching)
10	1221	C–C, C–O, C=O stretching in G ring
11	1160	Deformation vibrations of C–H bonds on benzene rings
12	1120	Carbon ring stretching of cellulose
13	1115	Vibrations of ester linkage
14	1086	C–O stretch of secondary alcohols and aliphatic ethers
15	1048	C–O stretch in cellulose and hemicellulose
16	1060/1040/1030	C–O stretch (primary alcohols)

Changes of the S and G units were also observed in the IR spectra. Peaks at 1325, 1244, and 1268–1270 cm^−1^ decreased in solid residues, demonstrating the release of the syringyl and guaiacyl derivatives into the liquid phase. These results were consistent with the observation by Py-GC/MS analysis in which the addition of dilute acid enhanced release of S and G units to aqueous phase. The FTIR results showed that more than 90 % of S and G units from biomass solids could be removed.

Peaks at 1375, 1120, and 1030–1086 cm^−1^ were assigned to bond stretching vibrations in cellulose and hemicellulose. The decrease of these peak intensities was attributed to the solubilization of cellulose and hemicellulose into the aqueous phase during pretreatment. Particularly, sample C presented almost a 90 % peak decrease in the carbohydrate region.

### Characterization of the RSL by GC/MS

GC/MS can identify lignin derivatives in soluble phase, but it is less sensitive to most oligomeric lignin polymers (>1000) because of their low volatility [37]. As discussed above, RSL showed only less than 20 and 5 % of the total yields of original lignin in water-only and dilute acid flowthrough pretreatments, respectively. However, profiles of lignin derivatives in the RSL phase can provide evidence of the lignin depolymerization mechanism.

In this study, the identified derivatives from water-only and 0.05 % (w/w) sulfuric acid pretreatments were mainly vanillin, benzaldehyde, hydroxybenzaldehyde, 2-methoxy-4-vinylphenol, 2,6-dimethoxy-phenol, 4-hydroxy-3,5-dimethoxy-benzaldehyde, and benzoic acids. These compounds indicated possible Cα oxidation and cleavage of β-O-4 linkages [38]. In addition, these products suggested that dehydroxylation or demethoxylation reactions were present in both flowthrough pretreatments, which is consistent with FTIR results. Other identified derivatives included, interestingly, the compounds: 2,2′-methylenebis[6-(1,1-dimethylethyl)-4-methylphenol], 4-(1,1,3,3-tetramethylbutyl)-phenol, and 2,6-bis(1,1-dimethylethyl)-4-methylphenol (butylated hydroxytoluene) (Additional file 1: Table S1).

### 2-D NMR of Ball-milled poplar lignin and RISL

**Fig. 7:**
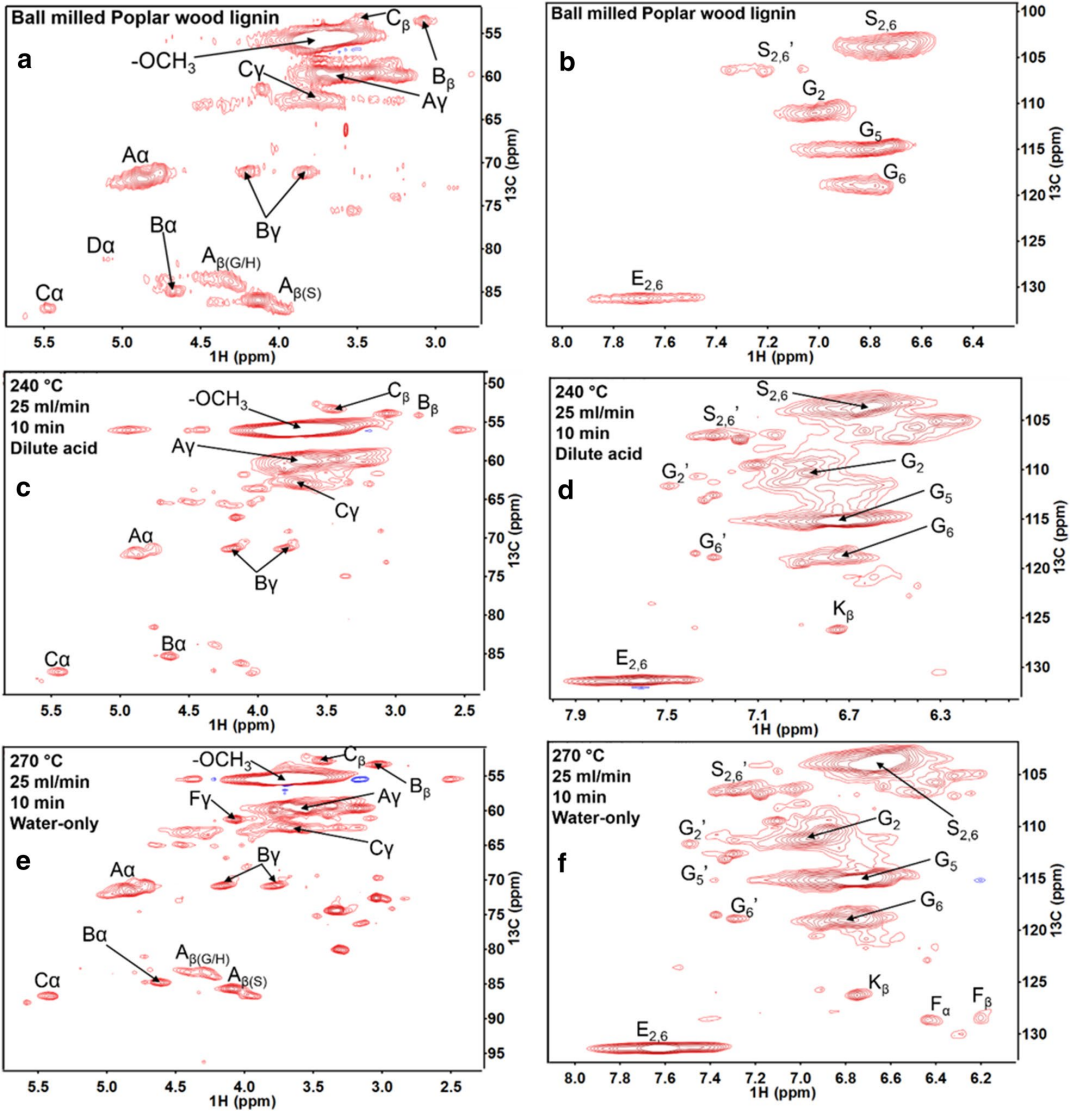
2-D ^1^H–^13^C HSQC correlation NMR spectra of aliphatic regions (*left column*) and aromatic regions (*right column)*. **a**, **b** Ball-milled poplar wood lignin; **c**, **d** 240 °C, 0.05 % (w/w) H_2_SO_4_ for 10 min (pretreatment severity ~ 5.0); **e**, **f** 270 °C, water-only for 10 min (pretreatment severity ~ 6.0); **a**: β–O–4 aryl ether linkages; **b**: resinol substructures (β-–β′, α–O–γ′, and γ–O–α′ linkages); **c**: phenylcoumaran substructures (β–5′ and α–O–4′ linkages); **d**: spirodienone substructures (β–1′ and α–O–α′ linkages); *G*: guaiacyl units; *G*′: oxidized guaiacyl units with a Cα ketone; *S*: syringyl units; *S*′: oxidized syringyl units with a Cα ketone; **e**: *p*-hydroxybenzoate substructures; **f**: cinnamyl alcohol end groups; *K*: cinnamaldehyde end groups. Their structures can be found in Additional file 1: Figure S2

Two-dimensional ^1^H–^13^C NMR (2-D NMR) provided evidence of lignin alteration by flowthrough pretreatments. Both the aliphatic (*δ*C/*δ*H 50–90/2.5–6.0 ppm) and aromatic (*δ*C/*δ*H 100–135/5.5–8.5 ppm) regions’ HSQC spectra were collected (Fig. 7). The assignments of the NMR signals are shown in Fig. 7 and summarized in detail in the supplemental material (Additional file 1: Table S2; Additional file 1: Figure S2). Relative peak area integrations for ball-milled lignin from the same poplar wood sample and RISLs were measured to determine each lignin linkage and monolignol change. The quantification of each linkage is from the volume integration of cross-peak contours in HSQC spectra according to previous publication [37]. Monolignol quantification was performed by peak integrations of peak C2/6 from S and H units and peak C2 from G units (doubled). The quantification of the basic linkages, Aα, Bα, and Cα contours were integrated to represent A, B, and C, respectively, among which Cα and Cβ contours in C represented α–O–4′ and β–5′ linkages [37, 39].

**Fig. 8:**
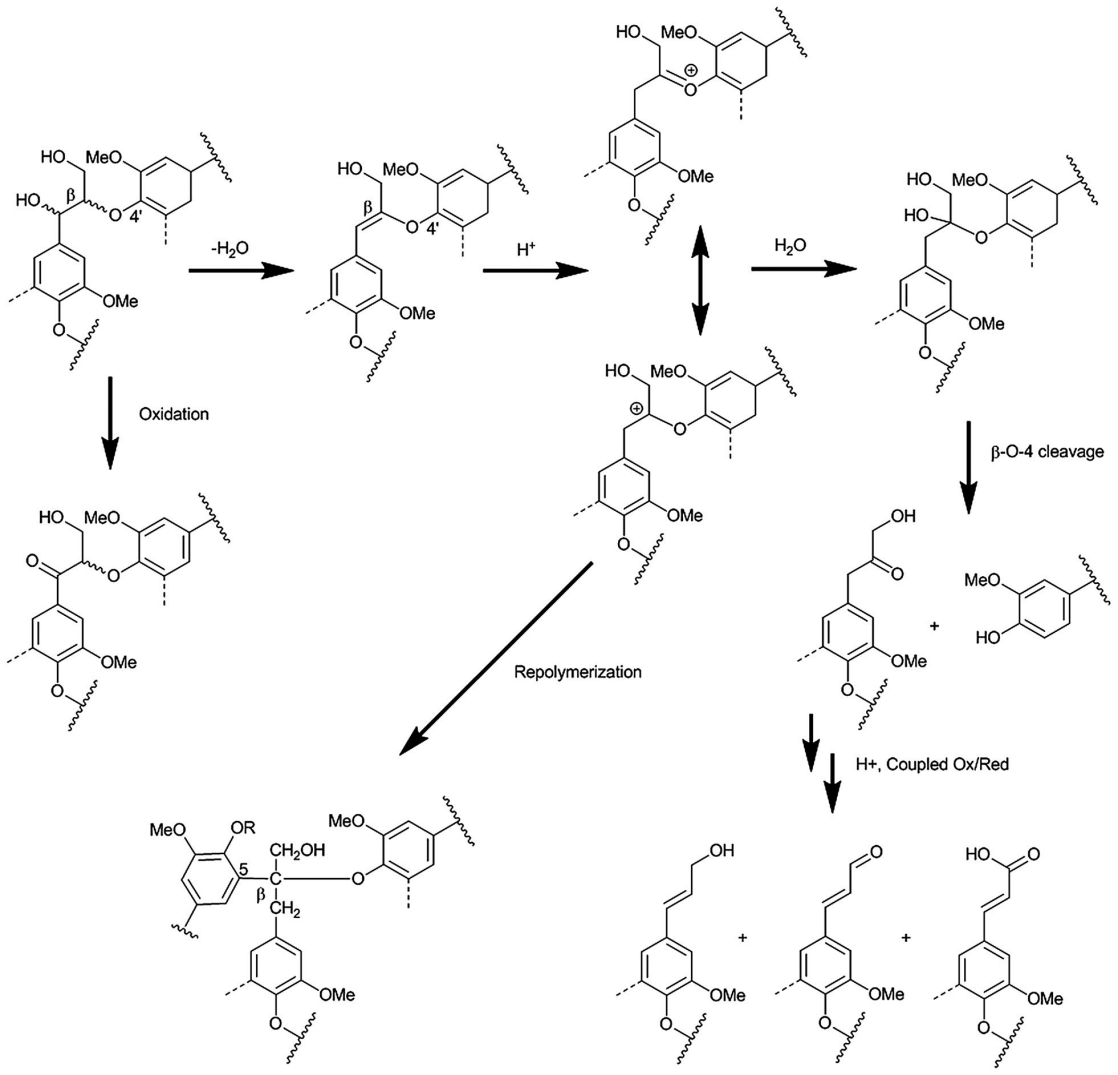
Plausible lignin β–O–4 cleavage and condensation reaction pathways in hydrolysates

The NMR spectra of pretreatment-derived lignins showed the basic linkages of lignin as in ball-milled wood lignin. Comparing pretreatment-derived lignin and ball-milled wood lignin indicates that flowthrough pretreatment only caused mild modification on original lignin. These structural changes include, first in the aliphatic region (in Fig. 7a, c, e), loss of structure D (spirodienone) which was not seen in both RISLs pretreated by water-only and dilute acid, indicating that flowthrough pretreatment decomposed this structure. Second, in the aromatic region, the RISL showed the formation of propenyl end group structures F (cinnamyl alcohol and its O4 ether and 3- and/or 5-methoxy analogs) and K (like cinnamaldehyde and analogs), which were not found in ball-milled poplar lignin. A plausible explanation for these propenyl end groups is β–O–4′ cleavage by a mechanism involving eliminative dehydration to remove the α-OH and produce an enol ether, which could then be cleaved by protonation of the enol, addition of water, and formation of a carbonyl at the α or β position upon cleavage of the aliphatic ether bond. Carbonyls at Cβ were not observed, while Cα carbonyls were observed, although either of these could be reduced and eliminated (dehydration) to yield the alkene. Hypothetically, oxidation of the γ-OH to the aldehyde or carboxylic acid could be a source of electrons for reduction of these carbonyls (Fig. 8).

Quantification of lignin linkages and substructures was made by 1-D ^13^C NMR spectra and by measuring contour volume integrals of 2-D HSQC peaks [39, 40]. The aromatic region provided S and G ratios in RISL, while the aliphatic region indicated an abundance of C–O–C and C–C linkages (Table 2). Ball-milled poplar lignin was mainly composed of β–O–4 ether, resinol, and phenylcoumaran structures with a small portion of spirodienone structure, *p*-hydroxybenzoate, and the *p*-hydroxycinnamyl alcohol. After pretreatment, the RISL showed different ratios of these linkages. However, the flowthrough pretreatment only caused a modest change in the ratio, especially for peak volumes in the aromatic region. First, the decline in percentages of β–O–4′ linkage, α–O–4′, and resinol structures indicated the depolymerization of these structures in the original lignin by pretreatment. Secondly, β–5′ structure slightly increased compared to ball-milled wood lignin indicating the occurrence of lignin β–5′ condensation reactions. The flowthrough system prevented severe condensation reactions that occur in batch reactors [41, 42]. Finally, cinnamyl-like structures appear in the NMR spectra, suggesting a β–O–4′ cleavage mechanism linked to dehydration at the α-position and oxidation at γ-OH. A possible mechanism is illustrated in Fig. 8.

**Table 2:** Relative proportions of major structural linkages and S, G, and H percentages in ball-milled lignin and flowthrough-derived RISL; A: RISL collected at 240 °C, 25 ml/min, 10 min, with 0.05 % (w/w) H_2_SO_4_; B: 270 °C, 25 ml/min, 10 min, with water-only

Samples	β–O–4	Resinol (β–β′)	Phenylcoumaran		Spirodienone (β–1′, α–O–α′)	S/G
			β–5′	α–O–4′		
Ball-milled	73.07	10.71	3.81	12.01	0.40	0.602
A	46.57	8.18	5.72	8.88	0.00	0.674
B	49.38	8.19	5.94	8.49	0.00	0.669

## Conclusions

Our previous work has shown great advantages in recovering sugars for our flowthrough pretreatment system operating at elevated temperature [25]. Understanding the fundamental chemistry of lignin during pretreatment is crucial to the development of advanced biorefinery strategies. In the flowthrough system, elevated temperature and addition of dilute acid were found to lead to cleavage of primarily C–O–C as well as C–C linkages in lignin, and effective solubilization of lignin derivatives into the aqueous phase with mild modification of lignin aromatic structures. Results indicated that 0.05 % (w/w) dilute sulfuric acid improved RISL yield as well as its purity. Together with elevated temperatures, 0.05 % (w/w) dilute sulfuric acid enhanced removal of not only S units but also most of G units, thus resulting in almost 100 % removal of lignin. Modest structural changes in side chains were observed, with formation of benzylic carbonyl groups at the α-position, as well as α–β unsaturation and oxidation of the γ-OH to the aldehyde. A hypothesized explanation for these propenyl end groups during the pretreatment is β–O–4′ aryl ether cleaved by dehydration at the α-position and oxidation at γ-OH. However, besides basic lignin linkages in RISL, such as resinol, β–O–4′, and the phenylcoumaran structures observed, slight repolymerization occurred in the form of new Cβ–C5´ linkages in RISL. Thus, flowthrough pretreatment of biomass has the potential to enable high-yield recovery of lignin derivatives for further upgrading, a potentially vital factor in the economics of biorefinery operations [1, 7, 15].

## Methods

### Materials

Hybrid poplar wood chips were kindly provided by the Forest Concepts Corporation, located in Auburn, Washington. The chips were air-dried and then milled to 0.4–0.8 mm. The resulting poplar particles were stored at −20 °C for experimental use with their moisture content at about 5 %. Compositions of poplar wood were determined using the NREL Laboratory Analytical Procedure (LAP; NREL, 2008) [43]. Poplar wood contains 48.5 ± 0.6 % glucan, 17.2 ± 0.5 % xylan, and 23.4 ± 0.4 % lignin. Ball-milled poplar lignin was kindly provided by Georgia Institute of Technology, Atlanta, Georgia.

### Flowthrough pretreatment of poplar wood

Poplar chips (0.5 g dry weight) were loaded into a tubular reactor (1.3 cm i.d. × 15.2 cm length with an internal volume of 20.5 ml). Two 5-µm silver-plated snubber gaskets were applied in both ends of the tubular reactor. The reactor was then connected to our flowthrough system. The flowthrough system was equipped with a 4-kW fluidized sand bath (model SBL-2D, Omega Engineering, Inc., Stamford, CT). Distilled water or 0.05 % (w/w) sulfuric acid at room temperature was pumped through a preheated coil in sand bath and then passed through the reactor continuously. The pretreatment hydrolysates were quenched by submerging the coil in ice water. The pretreatment was performed at 160–280 °C, and the temperature was monitored by a thermometer (Omega Engineering Co., Stamford, CT) located in the reactor outlet. The back pressure was regulated by a pressure gauge in the range of 10–70 bar, which corresponded to the saturated steam pressures. The sand bath temperature was controlled at 5–10 °C above the reaction temperature using a temperature controller (Omega, HH501AJK), with consideration of heat loss and transfer. The flow rate was set at 25 ml/min through the high-pressure pump (Acuflow Series III Pumps, Fisher, and Pittsburgh, PA, USA). After the pretreatment was completed, the reactor was immediately transferred to ice water. The undissolved poplar solid residues were collected and freeze-dried for analysis. In this study, the pretreatment severity factor was applied to evaluate pretreatment conditions (Eq. 1):

1 $${\text{Log }}R_{ 0} = {\text{Log }}t * {\text{exp}}\left(\frac{{T_{H} - T_{R} }}{ 1 4. 7 5} \right),$$

where *t* is the reaction time in minutes, *T*_*H*_ is the reaction temperature in °C, and *T*_*R*_ is the reference temperature in °C (100 °C) [44].

### Composition analysis of the solid residues and determination of lignin removal

Composition analysis of solid residues was carried out by the NREL procedure [43]. 0.03 g of solid residues underwent 72 % sulfuric acid hydrolysis in a 30 °C water bath for one hour, followed by 4 % sulfuric acid in autoclave with pressure at 121 °C for 1 h. The resulting slurries were filtrated and dried in the 105 °C oven. According to this method, the remaining oven-dried solids were ReL, known as acid insoluble lignin. Lignin removal refers to the percentage of original lignin released to aqueous phase (Eq. 2). This parameter presents the effectiveness of delignification by pretreatment.

2 $${\text{Lignin removal \% }} = 100 - \frac{{m_{\text{Re}} }}{{m_{\text{L}} }} \times 100,$$

where *m*_Re_ refers to the dry weight mass of remaining lignin after composition analysis of solid residues and *m*_L_ represents the mass weight of lignin in the original loaded biomass (*m*_L_ = the loaded biomass mass weight × the lignin content obtained from “Conclusions”).

### Ultraviolet–visible spectroscopy (UV–Vis) quantification of RSL

The RSL content (%) was determined by the UV absorbance measurement of the pretreated hydrolysates at 320 nm according to the NREL procedure [45]. RSL was calculated according to the following equations (Eqs. 3, 4).

3 $${\text{RSL\,\% }} = \frac{{{\text{UV}}_{\text{abs}} \times {\text{Volume}}_{\text{pretreatment liquor}} \times {\text{Dilution}}}}{{\varepsilon \times {\text{ODW}}_{\text{sample}} \times {\text{Pathlength}}}} \times 100,$$

where UV_abs_ is the average UV–Vis absorbance at 320 nm, ${\text{Volume}}_{\text{pretreatment liquor}}$ refers to the volume of pretreatment hydrolysate, *ɛ* represents the molar absorptivity of biomass at 320 nm (30 L/g cm), ODW_sample_ is the weight of sample in milligrams, and Pathlength is the pathlength of UV–Vis cell in cm.

4 $${\text{Dilution}} = \frac{{{\text{Volume}}_{\text{sample}} + {\text{Volume}}_{\text{diluting solvent}} }}{{{\text{Volume}}_{\text{sample}} }}$$

### Recovered insoluble lignin (RISL) isolation and purity measurement

The pH of some collected pretreatment hydrolysate samples that were not at pH 2–3 was adjusted to pH 2–3 to precipitate the RISL. The resulting precipitates were freeze-dried to obtain dry, solid RISL [1]. The RISL yield was calculated according to Eq. 5:

5 $${\text{RISL}}\,\% = \frac{{m_{\text{p}} }}{{m_{\text{L}} }} \times 100,$$

where *m*_p_ is the mass weight of precipitated lignin and *m*_*L*_ represents the mass weight of the lignin in the original loaded biomass (*m*_L_ = the loaded biomass mass weight × the lignin content calculated in “Conclusions”).

The RISL purity analysis was performed by determining the ultraviolet absorbance at 280 nm after subjecting the lignin sample to acetyl bromide (AcBr method), followed by acetic acid treatments [46]. The purity was calculated using the equation of Morrison (Eq. 6):

6 $${\text{AcBr lignin}} = \left(3.37 \times \frac{{U_{\text{abs}} }}{{C_{\text{L}} }} - 1.05\right) \times 100,$$

where *U*_abs_ refers to the UV absorbance at 280 nm and *C*_L_ is the lignin concentration before UV measurement.

### Gel permeation chromatography analysis of molecular weight of flowthrough lignin

The molecular weight analysis of the flowthrough-derived lignin was carried out using gel permeation chromatography (GPC). Lignin samples were individually acetylated by stirring with 2 ml of 1:1 acetic anhydride/pyridine (v/v) at room temperature for 24 h. After acetylation, the acetylated lignin samples were dissolved in THF for GPC analysis using an Agilent 1200 LC equipped with an ultraviolet (UV) detector. The sample was filtered through a 0.45 μm membrane filter prior to injection of a 20 μl sample. GPC analyses were carried out using a UV detector (280 nm) on a 4-column sequence of Waters TM Styragel columns (HR0.5, HR2, HR4, and HR6) at 1.00 ml/min flow rate. Polystyrene standards were used for calibration. WinGPC Unity software (Version 7.2.1, Polymer Standards Service USA, Inc.) was used to collect data and determine molecular weight profiles [1].

### Py-GC/MS analysis of ReL

Py-GC/MS was performed with a CDS 5000 pyrolysis autosampler (Oxford, PA, USA) attached to a Thermo Trace GC (6890 N/MSD, 5975B) gas chromatography/mass spectrometry system (Bellevue, WA, USA). Approximately 0.5 mg of the solid residues were loaded into a quartz tube that was filled with a thin layer of quartz wool in advance. The initial temperature maintained in the process was 250 °C for 6 s, and then the sample was pyrolyzed up to a temperature of 610 °C within 1 min. The generated vapor was separated by a 30 m, 0.25 μm inner diameter (5 % phenyl)-methylpolysiloxane column. The pyrolyzed gas was held in the pyrolysis oven for 56 min and then sent to the mass spectrometer that was operated in EI mode (70 eV) for analysis.

### Fourier transformed infrared (FTIR) spectroscopic analysis of ReL

The IR spectra (4500–800 cm^−1^) were obtained with a Bruker IFS 66v/s spectrometer equipped with an IR-microscope using about 2 mg of each sample. Spectra were obtained using the triangular apodization with a resolution of 4 cm^−1^ and an interval of 1 cm^−1^. 64 scans were conducted for each background and the sample spectra.

### Characterization of RSL after flowthrough pretreatment by GC/MS

10 ml of diethyl ether was used to extract RSL derivatives in 5 ml of hydrolysates, following vigorous mixing and subsequent suspension and separation. A 1 ml aliquot of the solvent phase was injected into an Agilent gas chromatograph mass spectrometer (GC/MS; GC, Agilent 7890A; MS, Agilent 5975C) equipped with a fused silica capillary column (DB-5MS column: 30 m × 320 µm × 0.25 µm). The carrier gas was helium at a flowrate of 1.3 ml/min. The splitter/injector was kept at 300 °C with a split ratio of 5:1. The oven temperature was programmed from 100 to 270 °C at a ramping rate of 5 °C/min. Both the initial and final temperatures were held for 5 min.

### RISL structural characterization by two-dimensional (2-D) heteronuclear single-quantum coherence (HSQC) solution nuclear magnetic resonance (NMR) spectroscopy

50 mg recovered insoluble lignin (RISL) was dissolved in 600 μl deuterated DMSO (Cambridge Isotope Laboratories). The resulting liquid sample was placed in 5-mm Wilmad 535-PP NMR tubes. NMR spectra were collected at 25 °C on 500 and 600 MHz Agilent (Varian) Inova NMR spectrometers equipped with *z*-axis pulsed-field gradient triple resonance HNCP probes. Samples contained 0.05 % (v/v) TMS for chemical shift referencing. Two-dimensional ^1^H–^13^C HSQC spectra of the aliphatic and aromatic regions were collected separately using the BioPack gchsqc pulse sequence, with ^1^H spectral width of 17 ppm and ^13^C spectral widths of 100 or 60 ppm for the aliphatic or aromatic regions, respectively. Spectra were collected with 1024 points (Varian parameter np) and 61 ms acquisition time with 128 or 256 transients and 128 or 96 complex points (Varian parameter ni in States-TPPI mode) in the indirect dimension, for aliphatic and aromatic spectra, respectively. Adiabatic WURST decoupling was applied during acquisition. Delayed times tCH and lambda for 1/4*J_CH_ were 1.8 ms and 1.6 ms for aliphatic spectra, and 1.45 ms and 1.3 ms for aromatic spectra, respectively. Reference one-dimensional ^1^H spectra were collected with 32 k points and 128 transients. HSQC spectra were processed and analyzed with Felix 2007 (FelixNMR, Inc) or MestReNova 6.0.4 (Mestrelab Research), with matched cosine-bell apodization in both dimensions, 2X zero filling in both dimensions, and forward linear prediction of 30 % more points in the indirect dimension. One-dimensional ^1^H spectra were processed with no apodization or linear prediction and 2× zero filling. Relative peak integrals were measured in MestReNova.

## Additional file

10.1186/s13068-015-0377-x Table S1. Major GC/MS detected aromatic compounds in hydrolysates. Table S2. Assignments of main lignin ^1^H-^13^C cross-peaks in the HSQC Spectra of the RISLs. Figure S1. Gel permeation chromatography (GPC) analysis of flowthrough lignin samples; Red: lignin obtained under 240 °C, residence time of 10 min, 0.05 % sulfuric acid and flow rate of 25 ml/min (Log R_0_ ~ 5.0); Blue: lignin obtained under 270 °C, residence time of 10 min, water-only and flow rate of 25 ml/min (Log R_0_ ~ 6.0). Figure S2. Assigned interunit linkages of lignin, including different side-chain linkages, and aromatic units: (A) β-O-4 aryl ether linkages; (B) resinol substructures (β-β´, α-O-γ´, and γ-O-α´ linkages); (C) phenylcoumaran substructures (β-5´ and α-O-4´ linkages); (D) spirodienone substructures (β-1´ and α-O-α´ linkages); (G) guaiacyl units; (G´) oxidized guaiacyl units with ketone at Cα; (S) syringyl units; (S´) oxidized syringyl units with a Cα ketone; (E) p-hydroxybenzoate substructures; (F) cinnamyl alcohol end groups; (K) cinnamaldehyde end groups.

## Abbreviations

ReL: residual lignin in pretreated solid residues
RISL: recovered insoluble lignin in pretreated liquid
RSL: recovered soluble lignin in pretreatment liquid
GC/MS: gas chromatography/mass spectrometry
HPLC: high-performance liquid chromatography
2-D HSQC NMR: two-dimensional heteronuclear single-quantum coherence solution nuclear magnetic resonance spectroscopy
FTIR: Fourier transformed infrared
Py-GC/MS: Pyrolysis–gas chromatography/mass spectrometry
AcBr: acetyl bromide
UV–Vis: ultraviolet visible spectroscopy
H units: *p*-hydroxyphenyl units
G units: guaiacyl units
S units: syringyl units
