# Supplementary material for: Characterization of lignin derived from water-only and dilute acid flowthrough pretreatment of poplar wood at elevated temperatures
Source: Biotechnol Biofuels. 2015 Dec 1;8:203. doi: 10.1186/s13068-015-0377-x (PMC4681453; doi:10.1186/s13068-015-0377-x)
Supplement: Supplementary file 1 — 10.1186/s13068-015-0377-x Table S1. Major GC/MS detected aromatic compounds in hydrolysates. Table S2. Assignments of main lignin 1H-13C cross-peaks in the HSQC Spectra of the RISLs. Figure S1. Gel permeation chromatography (GPC) analysis of flowthrough lignin samples; Red: lignin obtained under 240 °C, residence time of 10 min, 0.05 % sulfuric acid and flow rate of 25 ml/min (Log R0 ~ 5.0); Blue: lignin obtained under 270 °C, residence time of 10 min, water-only and flow rate of 25 ml/min (Log R0 ~ 6.0). Figure S2. Assigned interunit linkages of lignin, including different side-chain linkages, and aromatic units: (A) β-O-4 aryl ether linkages; (B) resinol substructures (β-β´, α-O-γ´, and γ-O-α´ linkages); (C) phenylcoumaran substructures (β-5´ and α-O-4´ linkages); (D) spirodienone substructures (β-1´ and α-O-α´ linkages); (G) guaiacyl units; (G´) oxidized guaiacyl units with ketone at Cα; (S) syringyl units; (S´) oxidized syringyl units with a Cα ketone; (E) p-hydroxybenzoate substructures; (F) cinnamyl alcohol end groups; (K) cinnamaldehyde end groups. [file 13068_2015_377_MOESM1_ESM.docx]

Characterization of Lignin Derived from Water-only and Dilute Acid Flowthrough Pretreatment of Poplar Wood at Elevated Temperatures

Libing Zhang ^1^, Lishi Yan ^1^, Zheming Wang ^2^, Dhrubojyoti D. Laskar ^1^, Marie S. Swita ^3^, John R. Cort ^2^*, and Bin Yang^1^*

**_________________________________________**

1 Bioproduct Sciences and Engineering Laboratory, Department of Biological Systems Engineering, Washington State University, Richland, WA 99354, USA.

2 Fundamental and Computational Sciences Directorate, Pacific Northwest National Laboratory, Richland, WA 99354, USA.

3 Bioproduct Sciences and Engineering Laboratory, Pacific Northwest National Laboratory, Richland, WA 99354, USA.

* Correspondence: Bin Yang [binyang@tricity.wsu.edu](mailto:binyang@tricity.wsu.edu); John R. Cort [John.Cort@pnnl.gov](mailto:John.Cort@pnnl.gov)

**Addition file**

**Table S1** Major GC/MS detected aromatic compounds in hydrolysates

| Major lignin derivatives | Relative abundance | | | | | |  | Structure | MW |
| --- | --- | --- | --- | --- | --- | --- | --- | --- | --- |
|  | 4.2^d^  ACID^a^ | 4.4^d^  ACID^a^ | 4.7^d^  ACID^a^ | 4.5^d^  HW^b^ | 6.0^d^  HW^b^ | 4.8^d^ ACID^c^ | 5.1 ^d^  ACID^c^ |  |  |
| Vanillin | + | - | - | - | - | + | + |  | 152 |
| Butylated Hydroxytoluene (BHT) | ++ | ++ | ++ | ++ | +++ | ++ | + |  | 220 |
| Phenol, 4-(1,1,3,3-tetramethylbutyl)- | + | + | +++ | + | ++ | +++ | +++ |  | 206 |
| Coniferyl alcohol | - | - | - | - | - | + | - |  | 180 |
| Phenol, 2,2'-methylenebis[6-(1,1-dimethylethyl)-4- | +++ | +++ | +++ | ++ | ++ | +++ | +++ |  | 340 |

**a**: 0.05%(w/w) H_2_SO_4_, 62.5mL/min; **b**: Water-only, 25mL/min; **c**: 0.05%(w/w) H_2_SO_4_, 25mL/min; **d**: pretreatment severity factor. + 0－10%; ++ 10%－20%; +++ 20%－50%; - not available.

**Table S2** Assignments of main lignin ^1^H- ^13^C cross-peaks in the HSQC Spectra of the RISLs [[1-6](#_ENREF_1)]

| Lignin linkages and monolignols | Chemical shift |
| --- | --- |
| -OCH_3_ | 55.47(C) 3.70(H) |
| A: β-O-4 | 71.70(S-Cα) 4.84(S-Hα) 59.50-59.70(Cγ) 3.40-3.63(Hγ) 85.90(S- Cβ) 4.09(S-Hβ) 83.49(G/H-Cβ) 4.28(G/H-Hβ) |
| B: resinol | 84.85(Cα) 4.62(Hα) 53.30(Cβ) 3.05(Hβ) 70.85(Cγ) 4.14/3.78(Hγ) |
| C: phenylcoumaran | 86.79(Cα) 5.41(Hα) 53.3(Cβ) 3.46(Hβ) 62.52(Cγ) 3.68(Hγ) |
| D: spirodienone | 59.67(Cβ) 3.19(Hβ) |
| G: guaiacyl | 111.02(C2) 6.95(H2) 115.05(G5) 6.74(H5) 119.01(G6) 6.78(H6) |
| G’: oxidized (Cα=O) guaiacyl | 111.56(C2) 7.50(H2) 123.55(C6) 7.54(H6) |
| S: syringyl | 103.95(C2/6) 6.67(H2/6) |
| S’: oxidized (Cα=O) syringyl | 106.52(C2/6) 7.29(H2/6) |
| E: *p*-hydroxybenzoate | 131.33(C2/6) 7.62(H2/6) |
| F: cinnamyl alcohol | 128.39(Cβ) 6.20(Hβ) 128.59(Cα) 6.42(Hα) |
| K: cinnamaldehyde | 126.23(Cβ) 6.74(Hβ) |

^a^ Note: G, S, H-C or G, S-H refers to C and H in the lignin sub-units, guaiacyl, syringyl and p-hydroxyphenyl.

**Figure S1** Gel permeation chromatography (GPC) analysis of flowthrough lignin samples; Red: lignin obtained under 240$℃$, residence time of 10mins, 0.05% sulfuric acid and flow rate of 25ml/min (LogR_0_ ~5.0); Blue: lignin obtained under 270$℃$, residence time of 10mins, water-only and flow rate of 25ml/min (LogR_0_ ~6.0)

**Figure S2** Assigned interunit linkages of lignin, including different side-chain linkages, and aromatic units: (A) β-O-4 aryl ether linkages; (B) resinol substructures (β-β′, α-O-γ′, and γ-O-α′ linkages); (C) phenylcoumarane substructures (β-5′and α-O-4′ linkages); (D) spirodienone substructures (β-1′ and α-O-α′ linkages); (G) guaiacyl units; (G′) oxidized guaiacyl units with an Cα-ketone; (S) syringyl units; (S′) oxidized syringyl units with a Cα ketone; (E) p-hydroxybenzoate substructures; (F) alcohol end groups; (K) cinnamaldehyde end groups.

**References**

1. Yuan TQ, Sun SN, Xu F, Sun RC: **Characterization of lignin structures and lignin-carbohydrate complex (LCC) linkages by quantitative 13C and 2D HSQC NMR spectroscopy**. *Journal of agricultural and food chemistry* 2011, **59**(19):10604-10614.

2. Pu Y, Hallac B, Ragauskas AJ: **Plant Biomass Characterization: Application of Solution‐and Solid‐State NMR Spectroscopy**. *Aqueous Pretreatment of Plant Biomass for Biological and Chemical Conversion to Fuels and Chemicals*:369-390.

3. Del Río JC, Rencoret J, Prinsen P, Martínez AnT, Ralph J, Gutiérrez A: **Structural characterization of wheat straw lignin as revealed by analytical pyrolysis, 2D-NMR, and reductive cleavage methods**. *Journal of agricultural and food chemistry* 2012, **60**(23):5922-5935.

4. Wen J-L, Sun S-L, Xue B-L, Sun R-C: **Recent advances in characterization of lignin polymer by solution-state nuclear magnetic resonance (NMR) methodology**. *Materials* 2013, **6**(1):359-391.

5. Ralph J, Akiyama T, Kim H, Lu F, Schatz PF, Marita JM, Ralph SA, Reddy MS, Chen F, Dixon RA: **Effects of coumarate 3-hydroxylase down-regulation on lignin structure**. *Journal of Biological Chemistry* 2006, **281**(13):8843-8853.

6. Rencoret J, Marques G, Gutierrez A, Ibarra D, Li J, Gellerstedt G, Santos JI, Jimenez-Barbero J, Martinez AT, del Rio JC: **Structural characterization of milled wood lignins from different eucalypt species**. *Holzforschung* 2008, **62**(5):514-526.
